# Understanding small Chinese cities as COVID-19 hotspots with an urban epidemic hazard index

**DOI:** 10.1038/s41598-021-94144-1

**Published:** 2021-07-19

**Authors:** Tianyi Li, Jiawen Luo, Cunrui Huang

**Affiliations:** 1 Department of Decision Sciences and Managerial Economics, CUHK Business School, Hong Kong, China; 2 Institute of Geophysics, ETH Zurich, Zurich, Switzerland; 3 Department of Health Policy and Management, School of Public Health, Sun Yat-sen University, Guangzhou, China; 4 Shanghai Key Laboratory of Meteorology and Health, Shanghai Meteorological Service, Shanghai, China; 5 School of Public Health, Zhengzhou University, Zhengzhou, China

**Keywords:** Infectious diseases, Applied mathematics, Software

## Abstract

Multiple small- to middle-scale cities, mostly located in northern China, became epidemic hotspots during the second wave of the spread of COVID-19 in early 2021. Despite qualitative discussions of potential social-economic causes, it remains unclear how this unordinary pattern could be substantiated with quantitative explanations. Through the development of an urban epidemic hazard index (EpiRank) for Chinese prefectural districts, we came up with a mathematical explanation for this phenomenon. The index is constructed via epidemic simulations on a multi-layer transportation network interconnecting local SEIR transmission dynamics, which characterizes intra- and inter-city population flow with a granular mathematical description. Essentially, we argue that these highlighted small towns possess greater epidemic hazards due to the combined effect of large local population and small inter-city transportation. The ratio of total population to population outflow could serve as an alternative city-specific indicator of such hazards, but its effectiveness is not as good as EpiRank, where contributions from other cities in determining a specific city’s epidemic hazard are captured via the network approach. Population alone and city GDP are not valid signals for this indication. The proposed index is applicable to different epidemic settings and can be useful for the risk assessment and response planning of urban epidemic hazards in China. The model framework is modularized and the analysis can be extended to other nations.

Despite the nation-wide successful implementation of control measures against COVID-19^1,2,3^, multiple small- to middle-scale cities in China (Chinese cities could be ranked at a level basis (e.g., https://baike.baidu.com) according to their development conditions; cities at or below the third level are normally referred to as small- to middle-scale (or peripheral; see *Model*)) became epidemic hotspots during the early-2021 wave of the pandemic; the list includes Tonghua, Songyuan, Suihua, Qiqihar, Heihe, and Xingtai etc.^4,5^. Unlike their nearby metropolis (e.g., Shijiazhuang, Changchun, Harbin; Chinese provincial capitals), these small towns are largely unknown to many Chinese before they are enlisted as “high-risk regions” after the local epidemic bursts, and it is indeed an unexpected phenomenon that these towns are highlighted among the over 300 Chinese prefectural administrations. It implies that the likelihood of epidemic hazards should be high in these regions. Many social-economic factors may account for this fact^6,7^: for example, social scientists may observe that these towns are all located in the northeast part of China, where local economies are often underdeveloped, and local residents are often more behavioral active than they are supposed to be in face of the epidemic (e.g.,^8^); other conjectures may attend to the fact that since these are neither coastal cities nor metropolitans where imported cases are more common, local control measures and regulations are thus somewhat relaxed in these regions, which led to heedlessness of early signals (e.g.,^9,10^), or that these northern regions have cold winters and also less residential housing space than the south, hence the hazard of severe infections was harbored (e.g.,^11^). Although these arguments are sound, it is desired that quantitative reasoning could be addressed to explain why these small Chinese towns stood out as epidemic hotspots.

This points to the significant necessity of the quantification of urban regions’ epidemic hazards, desirably via a constructed index that assesses the extent of potential risk. Such risk indices can be useful for effective decision analysis during epidemic response and planning^12,13^, critical to the mitigation of sudden and potentially catastrophic impacts of infectious diseases on society^14^. Indeed, although the exit of COVID-19 is still on the fly, both methodologically and practically^15^, it is nevertheless prudent to start getting prepared for the next pandemic^16,17,18^, both economically and ecologically^19,20^, through comprehensive pandemic risk management synthesis^21^ and the upgraded implementation of digital technologies^22^.

Many successful attempts have been made from various angles to develop such epidemic risk indices. According to US CDC, the preparedness for influenza pandemics can be assessed with the Influenza Risk Assessment Tool^23,24,25^ through the Pandemic Severity Assessment Framework^26,27^; for the same purpose, the Tool for Influenza Pandemic Risk Assessment^28^ is recommended by WHO; and there are miscellaneous other tools developed for national-level pandemic planning, through either mathematical simulations (e.g.,^29^) or scoring systems, in which various social-economic factors are considered (e.g.,^30,31,32^).

However, it is suggested that currently a suitable public health evaluation framework for the assessment of epidemic risk and response is still not full-fledge in scale^33^. Most proposed tools and frameworks are subject to a number of shortcomings: (1) assessments are in most cases from the supply side (i.e., the preparedness) instead of from the demand side (i.e., the actual risk); (2) assessments of pandemic potential are often virus-specific (i.e., pathological), while not as general-purpose as sufficiently considering important societal factors (such as transportation or population^34^); (3) many indices rely on expert scoring systems that often depend largely on subjectivity, and the calling for mathematical models and algorithms for risk assessment and pandemic planning is compelling^35^; (4) finally, many models focus on nation-wide evaluation, and there is relatively little concentration on sub-nation (e.g., city) level analysis, except for a few successful studies (e.g.,^36,37,38^).

To deal with these problems, in this study we develop a novel epidemic hazard index for Chinese cities, which quantifies the potential risk of epidemic spread for Chinese prefectural administrations (over 300 units). The index relies on a simulation model which integrates intra-city compartment dynamics with a detailed mathematical description of inter-city multi-channel transportation. Calculation of the hazard index is based on this dynamic system that simulates the domestic epidemic spread for user-specified diseases. In the model, intra-city evolution is governed by the SEIR dynamics, assuming no epidemic response taken place, such that the constructed index serves as an early-warning indicator at initial periods of epidemics, before the incidence of any structural change in the population flow upon policy intervention (e.g.,^39,40^); inter-city transportation is modeled with a multi-layer bipartite network^41^, which makes explicit considerations of various factors during inter-city population flow, including transit events, cross-infection due to path overlap, as well as the different transmissivities on different transportation media.

Such a highlight on transportation (i.e., spacial patterns) is core to the city-specific risk assessment of epidemics^42^ and natural hazards in general^43^. Indeed, over the course of the still on-going pandemic, it is acknowledged that transportation, at both the global and the domestic level, plays a critical role during the spread of viruses and to a large extent may determine the severity of the disease at different geological divisions^44,45^. Essentially, compared to regression models^46^ or the machine-learning approach^47^, the highlight of transportation asks for epidemic risk analysis from a network perspective (e.g.,^48,49,50,51^), upon which the risk scores could then be computed from quantitative approaches^52^. An important precedent is the Global Epidemic and Mobility (GLEaM) model, which integrates sociodemographic and population mobility data in a spatially structured stochastic disease approach to simulate the spread of epidemics at the worldwide scale^53^. GLEaM considers the commuting on the airport network on top of local disease transmission, where transportation is modeled via an effective operator; our model adopts a similar methodology yet constructs a more realistic multi-layer mathematical description of inter-city transportation, comparing also to various recent studies in the same line of research^54,55,56,57,58^.

## Data and methodology

### Model

The base model is developed in^41^ and is summarized here. Assume a bi-partite graph with cities (nodes) classified as either central cities or peripheral cities based on their development conditions (we regard central cities as level 0-2 cities and peripheral cities as small-to-middle scale cities/towns ranking at level three or below; see supplemental materials in^41^). The network is multi-layer $G = (V,E_{A/B/R/S})$, specifying four means of inter-city transportation (thus different layers have different edge connectivites between nodes): Air (A), Bus (B), Rail (R) and Sail (S). At each node, the local urban population is divided into four compartments: Susceptible (S), Exposed (E), Infected (I), Recovered (R), and the intra-city epidemic spread follows the standard SEIR dynamics (e.g.,^59,60^). We track the in- and out-flow of the exposed (E), susceptible (S) and recovered (R) population at each node on the inter-city transportation network, which determines the open-system SEIR dynamics (for a specific city *i*):

1 $$\begin{aligned} \left\{ \begin{array}{l} {\dot{S}}_i = - \frac{S_i}{P_i}\left(\frac{R_0}{D_I}I_i + z_i\right)+ \Delta S_i^{in} - \Delta S_i^{out}\\ {\dot{E}}_i = \frac{S_i}{P_i}\left(\frac{R_0}{D_I}I_i + z_i\right) - \frac{E_i}{D_E} + \Delta E_i^{in} - \Delta E_i^{out}\\ {\dot{I}}_i = \frac{E_i}{D_E} - \frac{I_i}{D_I} \\ {\dot{R}}_i = \frac{I_i}{D_I} + \Delta R_i^{in} - \Delta R_i^{out}. \end{array} \right. \end{aligned}$$

Epidemiological parameters $R_0$, $D_E$, $D_I$ are the basic reproduction number, the incubation period, and the infection period, respectively; $z_i=z_i(t)$ is the zoonotic force, i.e., the seed of the disease, with $z_i\ne 0$ only at node(s) that is(are) the disease epicenter(s). The in-flow and out-flow of each city are determined via the flowmaps $F^q = \{f_{i,j}^q\}$ between pairs of cities (*i*, *j*) specified for each means of transportation *q*. During inter-city population flow, transit events are considered, where only a certain proportion of flow (the proportion is $TR_{c/p}$ for central/peripheral cities, the same for each layer) enter the local population, and the rest are directed to other destinations. Moreover, cross-infections during inter-city travels are modeled, which take place between a susceptible person and an exposed person who share an overlapped travel path with the same destination. The strength of the cross-infection spillover $R_T^q$ varies on different transportation media (e.g.,^61^).

At each city *i*, the exposed inflow ($\Delta E_i^{in}(t)$) is given by

2 $$\begin{aligned} \Delta E_i^{in}(t) = \underset{q\in {Q}}{\sum } \underset{j\in {V}}{\sum } \overline{f_{j,i}^q (t) \mu _j (t)} (1-TR_i), \end{aligned}$$

where

3 $$\begin{aligned} \overline{f_{j,i}^q \mu _j}= f_{j,i}^q \mu _j + \underset{k}{\overset{p^q(i,k)\cap p^q(i,j)\ne 0}{\sum }} f_{k,i}^q \mu _k(R_T^q -1)\frac{f_{j,i}^q(1-\mu _j-\eta _j)\text {min}(d^q_{j,i},d^q_{k,i})}{\sum \nolimits _l^{{p^q}(i,k) \cap {p^q}(i,l) \ne 0} f_{l,i}^q (1-\mu _l-\eta _l)\text {min}(d^q_{l,i},d^q_{k,i})} \end{aligned}$$

is the adjusted exposed flow from city *j* to *i* by means *q*, taking care of cross-infections ($d^q_{i,j}$ represents the shortest path distance between *i* and *j* on layer *q*), and

4 $$\begin{aligned} \mu _i(t)= & {} \frac{\Delta E_i^{out}(t) + \underset{q\in {Q}}{\sum } \underset{j\in {V}}{\sum } \overline{f_{j,i}^q (t-1) \mu _j (t)} TR_i^q}{\underset{q\in {Q}}{\sum } \underset{j\in {V}}{\sum } f_{i,j}^q (t)}, \end{aligned}$$

5 $$\begin{aligned} \eta _i(t)= & {} \frac{\Delta R_i^{out}(t) + \underset{q\in {Q}}{\sum } \underset{j\in {V}}{\sum } f_{j,i}^q (t) \eta _j(t-1) TR_i^q}{\underset{q\in {Q}}{\sum } \underset{j\in {V}}{\sum } f_{i,j}^q (t)}, \end{aligned}$$

are the time-stamped proportion of the exposed and recovered population among the total outflow population from city *i*; thus $(1-\mu _i-\eta _i)$ is the proportion of the susceptible population among the total outflow from city *i*. The recovered inflow is tracking all the recovered people upon arrival (via $\eta _j$): $\Delta R_i^{in}(t) = \underset{q\in {Q}}{\sum } \underset{j\in {V}}{\sum } f_{j,i}^q (t) (1-TR_i^q) \eta _j(t-1)$, and according to flow balance, the susceptible inflow is $\Delta S_i^{in}(t) = \underset{q\in {Q}}{\sum } \underset{j\in {V}}{\sum } f_{j,i}^q (t) (1-TR_i^q) - \Delta E_i^{in}(t) - \Delta R_i^{in}(t)$. The outflow population from city *i*’s population $P_i$ is the total outbound flow minus the transferred inbound flow, contributed by the *S*, *E*, *R* compartments (with the proper assumption that *I* stay local, i.e., infected people do not participate in inter-city travels). Proportionally, the outflows are:

6 $$\begin{aligned} \Delta X_i^{out}(t) = X_i(t) \frac{ \underset{q\in {Q}}{\sum } \underset{j\in {V}}{\sum } f_{i,j}^q (t) - \underset{q\in {Q}}{\sum } \underset{j\in {V}}{\sum } f_{j,i}^q (t) TR_i^q}{S_i(t) + E_i(t) + R_i(t)}. \end{aligned}$$

with *X* being *S*, *E* or *R*.

**Figure 1:**
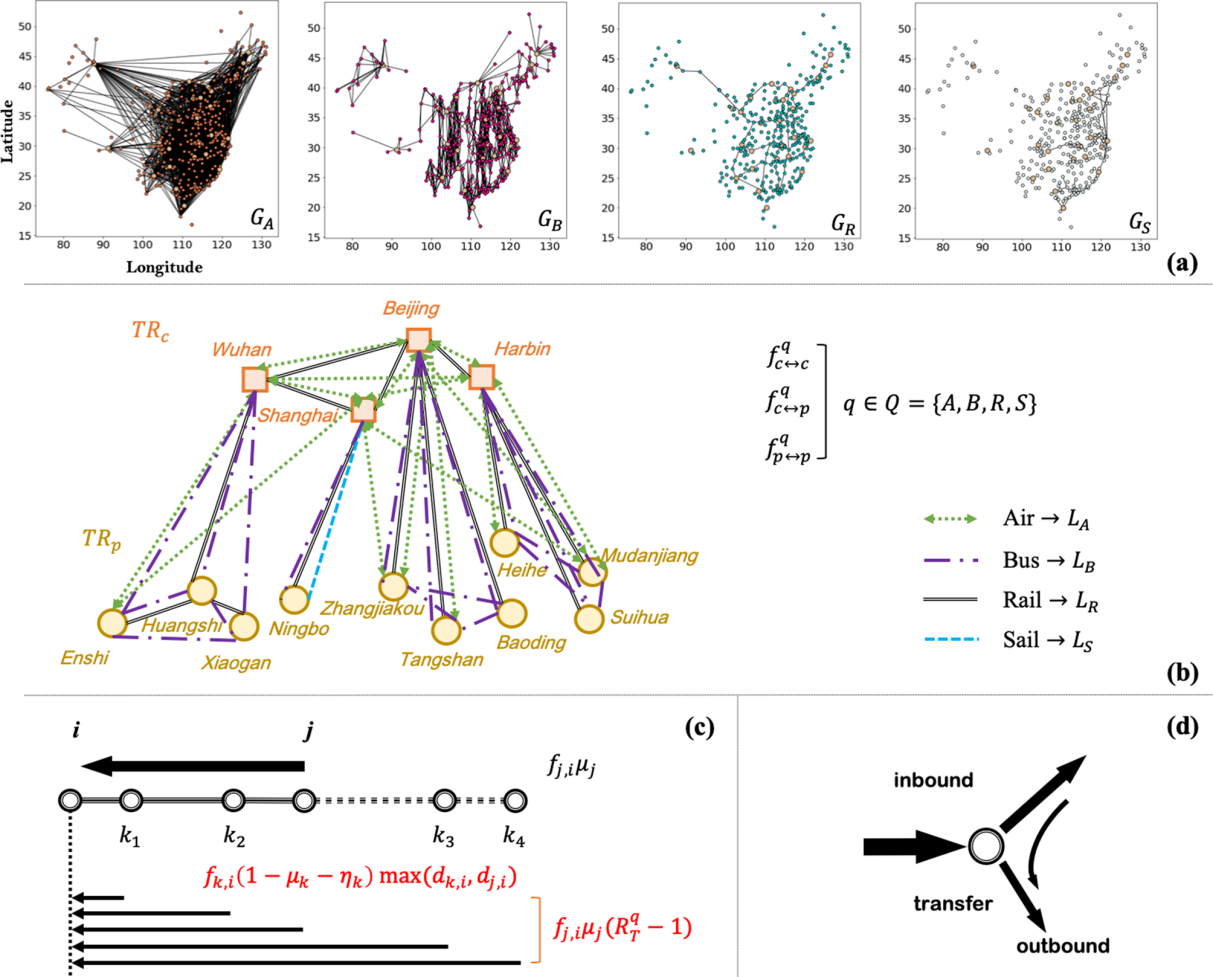
Multi-layer transportation network model with detailed flow description. (**a**) Four layers of inter-city transportation (Air, Bus, Rail, Sail) between Chinese prefectural administrations. (**b**) Bi-partite categorization of network nodes (central cities vs. peripheral cities). (**c**) Cross-infection during inter-city travel due to path overlap. (d) Inbound/outbound flow during transit.

Overall, the multi-layer network model is summarized in Fig. 1 (see more details in^41^).

### EpiRank

Using the constructed model framework, we are able to simulate the spread of imaginary diseases of arbitrary epidemiological features, originating from arbitrary epicenters. Suppose an epidemic initiating at node *i*, with a certain set of epidemiological parameters $R_0,D_E,D_I$. It is important to quantify the extent of its propagation on the domestic scale. This calls for a specific centrality measure of the epicenter node, which can characterize nodes’ strength in triggering epidemic spread. Borrowing from the idea of PageRank^62,63^, we construct this new centrality score in a way similar to the eigenvalue centrality and term it as *EpiRank*.

Start the simulator with a disease seeded at node *i* upon a given zoonotic force specified with *z*(*t*), which is non-zero during period $t_z^s$ to $t_z^e$. Consider a constant force $z_0$ over such a period (whose length is $\Delta t_z$), and the overall zoonotic force *Z* is:

7 $$\begin{aligned} Z = \int _{t_z^s}^{t_z^e} z(t) dt \sim \sum _{t_z^s}^{t_z^e} z(t) = (t_z^e - t_z^s)z_0 = \Delta t_z z_0. \end{aligned}$$

As the simulation proceeds, the local disease spreads to the entire nation along the transportation network. After $\tau$ time steps, we obtain the number of infected cases at city *j*, denoted as $I_{ij}^{\tau }$, with the first subscript indicating the epicenter. Similarly we obtain $R_{ij}^{\tau }$, $E_{ij}^{\tau }$ etc. We define the normalized total infection at city *j* as $U_{ij}^{\tau } = (I_{ij}^{\tau }+R_{ij}^{\tau })/Z$. $U_{ij}^{\tau }$ is then used to compute the EpiRank score for node *i* (denoted as $h_i^{\tau }$):

8 $$\begin{aligned} h_i^{\tau } = \alpha \underset{j\in {G},j\ne i}{\sum }\frac{f(U_{ij}^{\tau })}{\underset{j\in {G}}{\sum }f(U_{ij}^{\tau })} h_j^{\tau } + (1-\alpha )U_{ii}^{\tau }. \end{aligned}$$

$f(U_{ij}^{\tau })$ represents a specific function of $U_{ij}^{\tau }$ determining the relative weights of each city *j*’s score contributing to city *i*’s score. Here we simply allow $f(U_{ij}^{\tau }) = U_{ij}^{\tau }$, but further considerations could be made, such as applying a cutoff $U_0$ and $f(U_{ij}^{\tau }) = max(U_{ij}^{\tau }-U_0,0)$.

This score $h_i$ indicates the strength of epidemic spread at node *i*, which is contributed by (1) the city’s local severity of the epidemic, and (2) its ability of spreading the disease to other cities, with the EpiRank scores of other cities contributing to its own score at particular weights. $\alpha \in [0,1]$ is the parameter modulating the two effects: a small $\alpha$ (mode I) concerns heavier on the intra-city local spread of the epidemic ($\alpha = 0$ corresponds to a complete local index), while a large $\alpha$ (mode II) puts more weight on the consequences of epicenter’s spreading the disease to other regions.

Write $\varvec{W} = \{W_{ij}\}= \{U_{ij}^{\tau }/\underset{j\in {G}}{\sum }U_{ij}^{\tau }\}$; then $\varvec{h} = \{h_i^{\tau }\}$ can be calculated as follows:

9 $$\begin{aligned} \left\{ \begin{aligned}&h_i^{\tau } = \alpha \underset{j\in {G},j\ne i}{\sum } \frac{U_{ij}^{\tau }}{\underset{j\in {G}}{\sum }U_{ij}^{\tau }} h_j^{\tau } + (1-\alpha )U_{ii}^{\tau } \\&\Longrightarrow (1+ \alpha \frac{U_{ii}^{\tau }}{\underset{j\in {G}}{\sum }U_{ij}^{\tau }} )h_i^{\tau } = \alpha \underset{j\in {G}}{\sum } \frac{U_{ij}^{\tau }}{\underset{j\in {G}}{\sum }U_{ij}^{\tau }} h_j^{\tau } + (1-\alpha )U_{ii}^{\tau } \\&\Longrightarrow (1+ \alpha W_{ii})h_i^{\tau } = \alpha \underset{j\in {G}}{\sum } W_{ij} h_j^{\tau } + (1-\alpha )U_{ii}^{\tau } \\&\Longrightarrow [\varvec{I} -\alpha (\varvec{W} - \text {diag}(\varvec{W}))]\varvec{h} = (1-\alpha )\text {diag}(\varvec{U}) \mathbb {1} \\&\Longrightarrow \varvec{h} = (1-\alpha )[\varvec{I} -\alpha (\varvec{W} - \text {diag}(\varvec{W}))]^{-1}\text {diag}(\varvec{U}) \mathbb {1}. \\ \end{aligned} \right. \end{aligned}$$

When $\alpha = 0$, $\varvec{h} = \text {diag}(\varvec{U}) \mathbb {1}$. When $\alpha = 1$, $\varvec{h}$ is the eigenvector of [$\varvec{W} - \text {diag}(\varvec{W})$] of eigenvalue 1, in which case we may impose a value for $\text {max}(\varvec{h})$; wlog, we consider $\alpha \ne 1$. Note $\varvec{h} = \varvec{h}(\Delta t_z, z_0)$ but not $\varvec{h} = \varvec{h}(Z)$, since $\varvec{U} = \varvec{U}(\Delta t_z, z_0)$, i.e., the spread patterns may be different under different distributions of the same overall zoonotic force.

### Data and parameters

Connectivities of each network layer (i.e., transportation routes) are determined from public datasets and empirical considerations; city information (population, GDP etc.) is obtained from public datasets; transportation parameters (flowmap, transit rate, cross-infection strength) are determined through fitting the early spread of COVID-19 in China during January-February 2020, where the multi-parameter inversion is conducted via a smart gradient method (see^41^ and Supplemental Materials). Transit rate at central/peripheral cities is $TR_c = 0.4$ and $TR_p = 0.05$, respectively; cross-infection strength on the four transportation media is $\{R_T^A,R_T^B,R_T^R,R_T^S\} = \{1.2,3,1.5,1.5\}$; inter-city flows (elements of flowmaps) are different for different types of city pairs (central-central, central-peripheral, peripheral-peripheral), and are determined at $f_{cc/cp/pp}^A = 1000/500/0$, $f_{cc/cp/pp}^B = 0/3000/1000$, $f_{cc/cp/pp}^R = 2000/200/500$, $f_{cc/cp/pp}^S = 100/100/100$. These transportation parameters well fit the early spread of COVID-19 in China (^41^ and Supplemental Materials) and are fixed throughout the simulations.

For epidemiological parameters, the zoonotic force *z* is assumed to be 5 persons/day at Day 1 and zero afterwards, at a single epicenter (the simulator nevertheless allows for simultaneous bursts at multiple epicenters). The base-case disease is determined as $R_0 = 2.5$, $D_E = 6\ days$ and $D_I = 3\ days$, i.e., a mild reproduction of virus and a medium-range infection duration, close to the clinical features of COVID or SARS (e.g.,^64^). Unintervened spread of this seeded disease to all Chinese prefectural districts is simulated for 30 days, after which the ever-infected population ($I_i+R_i$) in each city *i* are recorded and are used to compute the hazard index $\varvec{h}$.

**Figure 2:**
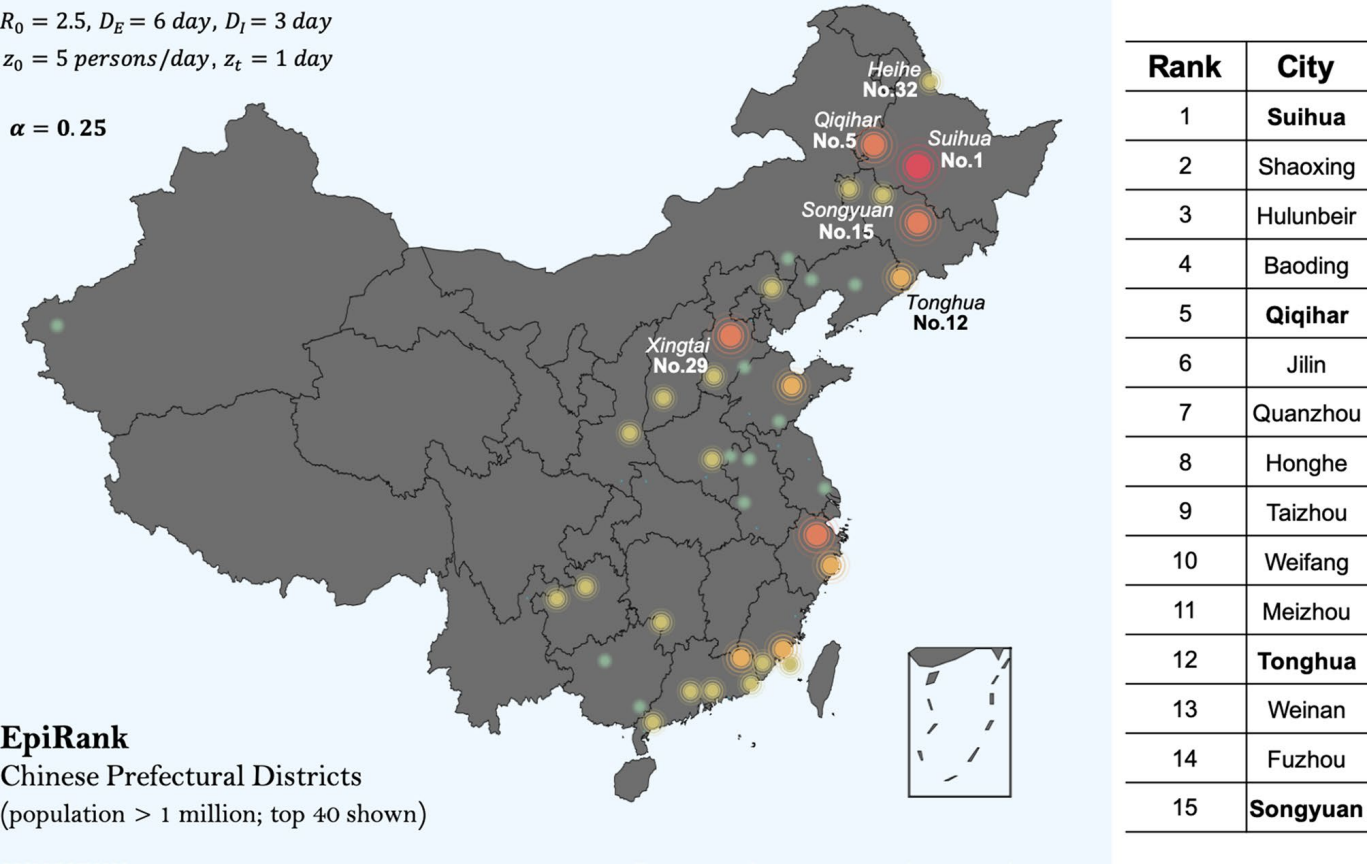
Urban Epidemic Hazard Index (EpiRank) of Chinese Cities (considering cities with population greater than 1 million; 300 units). Assuming a simulated disease with $R_0 = 2.5$, $D_E = 6\,\hbox {days}$, $D_I = 3\,\hbox {days}$, seeded by a 1-day zoonotic force at the strength of $5\,\hbox {persons/day}$; $\alpha =0.25$. Table: top 15 ranks. Six hotspots (Tonghua, Songyuan, Suihua, Qiqihar, Heihe, Xingtai) are successfully indicated by high rankings (four in top 5% and all six in top 10%). The map is generated using the python package pyecharts (http://pyecharts.herokuapp.com).

## Results

Cities having population larger than 1 million (300 units) participate in the calculation of $\varvec{h}$ under equation (9) (our method nevertheless applies to analysis on all >340 Chinese cities). For $\alpha =0.25$, the determined rankings of cities’ epidemic hazards (EpiRank) are shown in Fig. 2 (top 40 shown on the graph and top 15 listed in the table). One sees that, quite strikingly, the six small towns where the new bursts of COVID took place (Tonghua, Songyuan, Suihua, Qiqihar, Heihe, Xingtai) are successfully highlighted by the computed hazard index. All six cities rank within or near top 10% of all cities, including four cities ranking within top 5%. Tests suggest that the result is robust; the high ranks of the six highlighted cities are largely invariant to fluctuations in both transportation parameters and epidemiological parameters.

**Figure 3:**
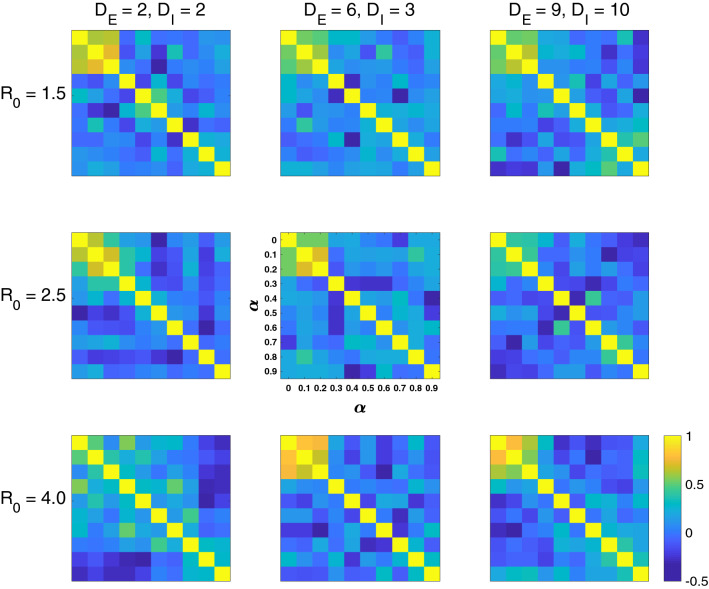
Spearman’s correlation of hazard rankings (top 30 entries in the rank) at different $\alpha$ (each sub-figure), for different sets of epidemiological parameters: $R_0 = 1.5/2.5/4$ (top to bottom) and infection duration $(D_E, D_I) = (2,2)/(6,3)/(9,10)$ days (left to right). Base case scenario (center) is $R_0 = 2.5$ and $(D_E, D_I) = (6,3)$.

The hazard rankings are computed at different $\alpha$, and correlation of the ranks is demonstrated via the Spearman’s correlation coefficient (Fig. 3; comparing top 30 entries of each rank). A stable ranking at small values of $\alpha$ is identified, along with a second invariant at the larger end (e.g., Fig. 3 top right). Indeed, the ranking is almost completely different at, for example, $\alpha = 0.1$ vs. $\alpha = 0.8$, with the latter having a new set of cities ranked top in the list, mostly located in the middle of China. This is consistent with our theory, as a small/large $\alpha$ points to either of the two end-members of epidemic hazards: the intra-city local spread vs. propagating the disease to domestic regions. Therefore, cities ranking high at small $\alpha$ (mode I) are regions with relatively large local population (since large local population $P_i$ substantiates large local infection $U_{ii}$ at city *i*) but also relatively small inter-city transportation (e.g., due to their rather insignificant geological positions or modest transportation infrastructures), in which case local epidemic bursts are severely harbored^65^ but not much spillovered to other cities. On the opposite, cities ranking high at large $\alpha$ (mode II) are regions where inter-city transportation is sufficiently viable (e.g., regions that are *carrefours* of transportation routes that have considerable access to greater domestic population) with respect to their relatively humble local population; in this case, when seeded a virus, the epicenter is less likely to become a closed epidemic cluster than to enormously propagate the disease to other regions.

We conducted simulations for different sets of epidemiological parameters of the assumed disease (Fig. 3), with the combination of low/medium/high infectivity ($R_0 = 1.5/2.5/4$) and short/medium/long infection duration ($(D_E, D_I) = (2,2)/(6,3)/(9,10)$ days). The invariant at small $\alpha$ (mode I) is largely maintained across the experiments, expect for a very severe disease with extremely high infectivity and extremely short duration ($R_0 = 4, (D_E, D_I) = (2,2)$). In this case, 30 days is sufficient for most population in most cities to get infected, and thus top rankings lean instead on densely populated cities. The second invariant at large $\alpha$ (mode II) is also identifiable, although not as clear as the first one. In some cases there is a third cluster at intermediate values of $\alpha$, but its significance is not as high as the first two. Overall, one is able to conclude that the two end-options of EpiRank, using small or large $\alpha$, lead to two converged outcomes across different scenarios of epidemic onset.

**Figure 4:**
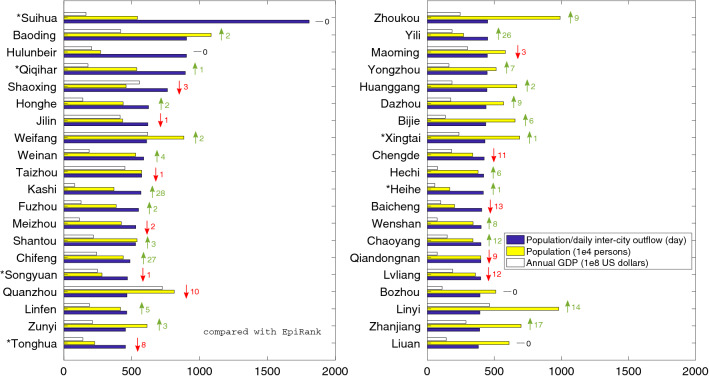
Local population/(daily) inter-city outflow as a simplified epidemic hazard indicator (showing top 40 cities in the ranking). Compared to EpiRank (results in Fig. 2), differences are marked with green, red or black arrows and numbers. This ratio is effective but less accurate than EpiRank in highlighting the six epicenters (with star marks). Shear population or annual GDP does not provide similar indication.

## Discussion

As mentioned, from the model and results one deduces that, the high epidemic hazard of these small towns, obtained at low $\alpha$ in which case $h_i$ draws heavily on city *i*’s own infection $U_{ii}$, derives from the combined effect of two elements: a relatively large local population that triggers substantial local infection, and a relatively small inter-city transportation flow determined by geological and infrastructural factors. Intuitively, a serious epidemic cluster at the city scale is going to develop, when the city is sufficiently populated, while not much inter-city outflow is dispersing the infection out of the epicenter. This inspires the idea that alternatively, we can compute the population/inter-city outflow ratio of each city and use this value to indicate cities’ epidemic hazard. Results (Fig. 4) show that similar to EpiRank, this ratio does serve as a decent hazard indicator, through which the six epicenters are listed with high ranks. Furthermore, by contrast, shear population and annual GDP, arguably two most considered social-economic indicators of urban regions^6^, are not helpful in revealing the epidemic ground-truth. Conceptually, analysis on EpiRank help us pin down the ratio of local population/inter-city outflow as a critical social-economic factor in explaining the observed phenomenon. For robust tests, we proportionally increased and decreased values on the flowmaps; results suggest that the effectiveness of this ratio (and certainly the effectiveness of EpiRank) in highlighting the six epicenters is largely invariant to changes in absolute flow strength.

Nevertheless, it is seen that this simple ratio, although still effective and easy to compute, is not as accurate and informative as EpiRank (Fig. 4). This time the six towns are overall lower ranked, with only 2 out of 6 in top 5%. This is because this ratio only considers a city’s own population and transportation condition, whereas EpiRank takes a full account of the regional and then the entire national picture, under the networked dynamics approach. Indeed, it is not empirically inconsistent to argue that the six epicenters are all located in the north, not only because they themselves have large population and small inter-city outflow, but also because it is exactly the case that cities in northern China, with which the six towns exchange most population, all tend to have such features and therefore the effect of local clusters is further locked in. The advantage of EpiRank is implied; certainly, the simple ratio is also not able to reveal the extent of epicenters’ spreading the disease out, as EpiRank can shed light on with high $\alpha$.

The model framework and the EpiRank index can be used for the analysis of other nations, presumably with a different set of transportation layers and corresponding datasets of flowmaps (e.g., for the US)^54^. However, although providing a promising quantitative explanation for the researched phenomenon, it is indiscreet to conclude that EpiRank is by any means a sufficiently accurate index of urban epidemic hazards. The current dynamic network model draws little besides the two aspects, urban population and inter-city transportation, and too many real-world factors are left out. More importantly, a major limitation of the current analysis is that the validation of EpiRank results is difficult to be conducted in a systematic and rigorous manner, besides using the six epicenters as the ground truth. Indeed, the ranking is about the *risk* (i.e., what is likely to happen), which is not always leading to the ground truth (i.e., what did happen). Cities ranking high in the index possess great risk to be epidemic hotspots, but the bursts may or may not turn out, and we are not able to conduct social experiments to carry out real-world tests on this topic. Furthermore, the current assumption that infected people stay local largely holds true in the Chinese case, but may fail in the general sense since asymptomatic people may not be diagnosed in time and may still participate in inter-city transportation, in which case cross-infection could be more serious. Since this factor influences all transportation flows in a proportional manner, our current normalized formulation is not substantially biased; nevertheless, in future extensions, advanced SEIR compartment models could be adopted, in which the asymptomatic stock can be separated from the infected stock, and the former group can remain active in public transportation. Overall, despite a mathematically consistent and empirically effective approach, the proposed simulation framework and the constructed EpiRank index call for extensive tests and advancements in various settings, before their power as well as shortcomings can be substantially uncovered; this study serves as a first attempt.

## Conclusion

In this study, we developed an index (EpiRank) to evaluate the urban epidemic hazard of Chinese cities via networked dynamic simulations. The index can be used in two functioning modes, and in one usage mode the results helped provide a quantitative explanation for the phenomenon that multiple small-to-middle scale cities in China became COVID-19 hotspots during the early-2021 wave. Through the analysis, we propose that the combined effect of large local population and small inter-city transportation at the highlighted epicenters could be the potential driving factor of this phenomenon. The model framework can be adapted to other nations, and extensive assessments of EpiRank are to be conducted further.

## Supplementary Information

Supplementary Information.

## Data Availability

Edge connectivities of transportation layers and information of Chinese prefectural administrations (population, GDP etc.) are collected from public datasets and available at https://github.com/TimothyLi0123/WH. COVID-19 datasets used in parameter estimation are from the public repository at https://github.com/BlankerL/DXY-COVID-19-Data. Scripts (python package) are available at https://github.com/TimothyLi0123/EpiRank.
