# Supplementary material for: Understanding small Chinese cities as COVID-19 hotspots with an urban epidemic hazard index
Source: Sci Rep. 2021 Jul 19;11:14663. doi: 10.1038/s41598-021-94144-1 (PMC8290012; doi:10.1038/s41598-021-94144-1)
Supplement: Supplementary file 1 — Supplementary Information. [file 41598_2021_94144_MOESM1_ESM.pdf]

Supplemental Materials for "Understanding Small Chinese Cities as COVID-19 Hotspots With An Urban Epidemic Hazard Index"

Tianyi Li

Department of Decision Sciences and Managerial Economics, CUHK Business School

Jiawen Luo

Institute of Geophysics, ETH Zurich

Cunrui Huang

Department of Health Policy & Management, School of Public Health, Sun Yat-sen University

Shanghai Key Laboratory of Meteorology and Health, Shanghai Meteorological Service

School of Public Health, Zhengzhou University

Model Summary

Let $S_i^{(n)}$, $E_i^{(n)}$, $I_i^{(n)}$ and $R_i^{(n)}$ be the number of Susceptible, Expose, Infected and Recovered people in city i and at time step n, respectively; the total population $P_i^{(n)} = S_i^{(n)} + E_i^{(n)} + I_i^{(n)} + R_i^{(n)}$. With a single Euler step approximation (which can be verified a posteriori by comparing forward model results), the open-system SEIR model can be described as

$$
\begin{aligned}
S_i^{(n+1)} &= S_i^{(n)} - \frac{S_i^{(n)}}{P_i^{(n)}}\left(\frac{R_0}{D_I} I_i^{(n)} + z_i^{(n)}\right) + \Delta S_i^{in} - \Delta S_i^{out} \\
E_i^{(n+1)} &= E_i^{(n)} + \frac{S_i^{(n)}}{P_i^{(n)}}\left(\frac{R_0}{D_I} I_i^{(n)} + z_i^{(n)}\right) - \frac{E_i^{(n)}}{D_E} + \Delta E_i^{in} - \Delta E_i^{out} \\
I_i^{(n+1)} &= I_i^{(n)} + \frac{E_i^{(n)}}{D_E} - \frac{I_i^{(n)}}{D_I} \\
R_i^{(n+1)} &= R_i^{(n)} + \frac{I_i^{(n)}}{D_I} + \Delta R_i^{in} - \Delta R_i^{out}.
\end{aligned}
\tag{1}
$$

R_0, D_E and D_I are the basic reproduction number, the incubation period, and the infection period, respectively; z is the zoonotic force. $\Delta S_i^{in}/\Delta S_i^{out}$, $\Delta E_i^{in}/\Delta E_i^{out}$ and $\Delta R_i^{in}/\Delta R_i^{out}$ represent inflow/outflow of compartments S, E, R at city i due to inter-city transportation. Let $\mu_i^{(n)}$ and $\eta_i^{(n)}$ be the proportion of the exposed and recovered population among the total outflow population of city i at time step n. They are computed as

$$
\begin{aligned}
\mu_i^{(n+1)} &= \frac{\Delta E_i^{out} + TR_i \sum_{q,j} f_{j,i}^q \mu_j^{(n)}}{\sum_{q,j} f_{i,j}^q} \\
\eta_i^{(n+1)} &= \frac{\Delta R_i^{out} + TR_i \sum_{q,j} f_{j,i}^q \eta_j^{(n)}}{\sum_{q,j} f_{i,j}^q},
\end{aligned}
\tag{2}
$$

where TR_i is the transfer rate at city i, and $f_{j,i}^q$ is the flow strength from city j to city i via transportation q. At a given time step n, to compute S, E, I, R, μ and η at time step $n+1$, we need to compute ΔS_i^{in},

ΔE_i^{in}, ΔR_i^{in}, ΔS_i^{out}, ΔE_i^{out} and ΔR_i^{out}. They are defined as

$$\begin{aligned}
\Delta\Theta_i^{out} &= \frac{\Theta_i}{S_i+E_i+R_i}\left[\sum_{q,j} f_{i,j}^q - TR_i \sum_{q,j} f_{j,i}^q\right], \quad \Theta = S,E,R \\
\Delta R_i^{in} &= (1-TR_i)\sum_{q,j} f_{j,i}^q \eta_j \\
\Delta E_i^{in} &= (1-TR_i)\sum_{q,j} \overline{f_{j,i}^q \mu_j} = (1-TR_i)\sum_q \alpha_i^q \\
\Delta S_i^{in} &= (1-TR_i)\sum_{q,j} f_{j,i}^q - \Delta E_i^{in} - \Delta R_i^{in},
\end{aligned} \tag{3}$$

where $\alpha_i^q = \sum_j \overline{f_{j,i}^q \mu_j}$ is the modified number of the exposed compartment due to cross-infection.

$$\overline{f_{j,i}^q \mu_j} = f_{j,i}^q \mu_j + \sum_{k \in p^q(i,k)\cap p^q(i,j)} f_{k,i}^q \mu_k \beta_q \frac{f_{j,i}^q (1-\mu_j-\eta_j)\min(d_{j,i}^q, d_{k,i}^q)}{\sum_{l \in p^q(i,k)\cap p^q(i,l)} f_{l,i}^q \min(d_{l,i}^q, d_{k,i}^q)}, \tag{4}$$

where β_q is the reproduction rate on transportation q. For end-to-end transportation, air (A) and bus (B), this formula simplifies to

$$\overline{f_{j,i}^q \mu_j} = f_{j,i}^q \mu_j + f_{j,i}^q \mu_j (1-\mu_j-\eta_j)\beta_q. \tag{5}$$

Model Parameters

We summarize the parameters that are to be estimated. Epidemiological parameters R_0, D_I,; D_E; TR_γ, $\gamma \in \{c,p\}$, is the transfer rate for central cities V_c and peripheral cities V_p; β_q is the reproduction rate on transportation q; $F_{\gamma\gamma'}^q$, $\gamma,\gamma' \in \{c,p\}$ represents the flow strength between γ type and γ' type cites on q. A constant zoonotic force z is assumed, with a priori duration m(days), its strength being the parameter. So we have

$$TR_i = \begin{cases} TR_c & i \in V_c \\ TR_p & i \in V_p \end{cases} \tag{6}$$

and the flow matrix can be written as

$$\mathbf{f}^q = F_{cc}^q \mathbf{f}^{q,cc} + F_{cp}^q \mathbf{f}^{q,cp} + F_{pp}^q \mathbf{f}^{q,pp}, \tag{7}$$

where $\mathbf{f}_{\gamma\gamma'}$ embeds the network structure and can be viewed as boolean matrices.

Computing Gradients

Write the misfit function as

$$\mathcal{L} = \mathcal{L}\left(S_i^{(n)}, E_i^{(n)}, I_i^{(n)}, R_i^{(n)}\right),$$

note that in general it is allowed to depend on values at all time steps. Let χ be a particular parameter of interest, and assume $\mathcal{L}$ being at least C^1, then $\partial\mathcal{L}/\partial\chi$ can easily be computed if $\partial S_i^{(n)}/\partial\chi$, $\partial E_i^{(n)}/\partial\chi$, $\partial I_i^{(n)}/\partial\chi$, and $\partial R_i^{(n)}/\partial\chi$ are known. The gradient will be computed inductively on time steps n. At current time step n, we compute the gradient for values at time step $n+1$. The process is done in the following order. For $\Theta = S,E,R$,

$$\frac{\partial\Delta\Theta_i^{out}}{\partial\chi} = \left(\frac{\partial\Theta_i/\partial\chi}{S_i+E_i+R_i} - \frac{\Theta_i \partial\Theta_i/\partial\chi}{(S_i+E_i+R_i)^2}\right)\left[\sum_{q,j} f_{i,j}^q - TR_i \sum_{q,j} f_{j,i}^q\right], \quad \chi = z, R_0, D_I, D_E, \beta_q, \tag{8}$$

$$\begin{aligned}\frac{\partial \Delta\Theta_i^{out}}{\partial TR_\gamma} &= \left(\frac{\partial\Theta_i/\partial TR_\gamma}{S_i+E_i+R_i} - \frac{\Theta_i\partial\Theta_i/\partial TR_\gamma}{(S_i+E_i+R_i)^2}\right)\left[\sum_{q,j} f_{i,j}^q - TR_i\sum_{q,j} f_{j,i}^q\right] \\ &\quad - (i\in V_\gamma)\,\frac{\Theta_i}{S_i+E_i+R_i}\sum_{q,j} f_{j,i}^q,\end{aligned} \tag{9}$$

$$\begin{aligned}\frac{\partial \Delta\Theta_i^{out}}{\partial F_{\gamma\gamma'}^q} &= \left(\frac{\partial\Theta_i/\partial F_{\gamma\gamma'}^q}{S_i+E_i+R_i} - \frac{\Theta_i\partial\Theta_i/\partial F_{\gamma\gamma'}^q}{(S_i+E_i+R_i)^2}\right)\left[\sum_{q,j} f_{i,j}^q - TR_i\sum_{q,j} f_{j,i}^q\right] \\ &\quad + \frac{\Theta_i}{S_i+E_i+R_i}\left[\sum_j f_{i,j}^{q,\gamma\gamma'} - TR_i\sum_j f_{j,i}^{q,\gamma\gamma'}\right],\end{aligned} \tag{10}$$

for $\xi=\mu,\eta$, and correspondingly $\Theta=E,R$

$$\frac{\partial\xi_i^{(n+1)}}{\partial\chi} = \frac{\partial\Delta\Theta_i^{out}/\partial\chi + TR_i\sum\limits_{q,j} f_{j,i}^q\partial\xi_j^{(n)}/\partial\chi}{\sum\limits_{q,j} f_{i,j}^q}, \quad \chi = z, R_0, D_I, D_E, \beta_q, \tag{11}$$

$$\frac{\partial\xi_i^{(n+1)}}{\partial TR_\gamma} = \frac{\partial\Delta\Theta_i^{out}/\partial TR_\gamma + TR_i\sum\limits_{q,j} f_{j,i}^q\partial\xi_j^{(n)}/\partial TR_\gamma}{\sum\limits_{q,j} f_{i,j}^q} + \frac{(i\in V_\gamma)\sum\limits_{q,j} f_{j,i}^q\xi_j^{(n)}}{\sum\limits_{q,j} f_{i,j}^q}, \tag{12}$$

$$\begin{aligned}\frac{\partial\xi_i^{(n+1)}}{\partial F_{\gamma\gamma'}^q} &= \frac{\partial\Delta\Theta_i^{out}/\partial F_{\gamma\gamma'}^q + TR_i\sum\limits_{q,j} f_{j,i}^q\partial\xi_j^{(n)}/\partial F_{\gamma\gamma'}^q + TR_i\sum\limits_j f_{j,i}^{q,\gamma\gamma'}\xi_j^{(n)}}{\sum\limits_{q,j} f_{i,j}^q} \\ &\quad - \frac{\Delta\Theta_i^{out} + TR_i\sum\limits_{q,j} f_{j,i}^q\xi_j^{(n)}}{\left(\sum\limits_{q,j} f_{i,j}^q\right)^2}\sum_j f_{i,j}^{q,\gamma\gamma'}.\end{aligned} \tag{13}$$

Next we compute the gradients for α_i^p, which is the heaviest part. For $p=0,3$, i.e. air or bus layer:

$$\begin{aligned}\frac{\partial\alpha_i^p}{\partial\chi} &= \sum_j f_{j,i}^p\partial\mu_j/\partial\chi + \beta_p\sum_j f_{j,i}^p\left[(1-\mu_j-\eta_j)\partial\mu_j/\partial\chi - \mu_j\left(\partial\mu_j/\partial\chi + \partial\eta_j/\partial\chi\right)\right], \\ &\qquad \chi = TR_\gamma, z, R_0, D_I, D_E,\end{aligned} \tag{14}$$

$$\begin{aligned}\frac{\partial\alpha_i^p}{\partial\beta^q} &= \sum_j f_{j,i}^p\partial\mu_j/\partial\beta^q + \beta_p\sum_j f_{j,i}^p\left[(1-\mu_j-\eta_j)\partial\mu_j/\partial\beta^q - \mu_j\left(\partial\mu_j/\partial\beta^q + \partial\eta_j/\partial\beta^q\right)\right] \\ &\qquad + \delta_{pq}\sum_j f_{j,i}^q\mu_j(1-\mu_j-\eta_j),\end{aligned} \tag{15}$$

$$\begin{aligned}\frac{\partial\alpha_i^p}{\partial F_{\gamma\gamma'}^q} &= \sum_j f_{j,i}^p\partial\mu_j/\partial F_{\gamma\gamma'}^q + \beta_p\sum_j f_{j,i}^p\left[(1-\mu_j-\eta_j)\partial\mu_j/\partial F_{\gamma\gamma'}^q - \mu_j\left(\partial\mu_j/\partial F_{\gamma\gamma'}^q + \partial\eta_j/\partial F_{\gamma\gamma'}^q\right)\right] \\ &\qquad + \delta_{pq}\left[\sum_j f_{j,i}^{q,\gamma\gamma'}\mu_j + \beta_q\sum_j f_{j,i}^{q,\gamma\gamma'}\mu_j(1-\mu_j-\eta_j)\right].\end{aligned} \tag{16}$$

For $p = 1, 2$, i.e. rail or sail layer:

$$\frac{\partial \alpha_i^p}{\partial \chi} = \sum_j \{ f_{j,i}^p \partial \mu_j / \partial \chi + \sum_{k \in p^p(i,k) \cap p^p(i,j)} f_{k,i}^p (\partial \mu_k / \partial \chi) \beta_p \frac{f_{j,i}^p (1 - \mu_j - \eta_j) \min(d_{j,i}^p, d_{k,i}^p)}{\sum_{l \in p^p(i,k) \cap p^p(i,l)} f_{l,i}^p \min(d_{l,i}^p, d_{k,i}^p)}$$
$$- \sum_{k \in p^p(i,k) \cap p^p(i,j)} f_{k,i}^p \mu_k \beta_p \frac{f_{j,i}^p (\partial \mu_j / \partial \chi + \partial \eta_j / \partial \chi) \min(d_{j,i}^p, d_{k,i}^p)}{\sum_{l \in p^p(i,k) \cap p^p(i,l)} f_{l,i}^p \min(d_{l,i}^p, d_{k,i}^p)} \}, \tag{17}$$
$$\chi = TR_\gamma, z, R_0, D_I, D_E,$$

$$\frac{\partial \alpha_i^p}{\partial \beta_q} = \sum_j \{ f_{j,i}^p \partial \mu_j / \partial \beta_q + \sum_{k \in p^p(i,k) \cap p^p(i,j)} f_{k,i}^p (\partial \mu_k / \partial \beta_q) \beta_p \frac{f_{j,i}^p (1 - \mu_j - \eta_j) \min(d_{j,i}^p, d_{k,i}^p)}{\sum_{l \in p^p(i,k) \cap p^p(i,l)} f_{l,i}^p \min(d_{l,i}^p, d_{k,i}^p)}$$
$$- \sum_{k \in p^p(i,k) \cap p^p(i,j)} f_{k,i}^p \mu_k \beta_p \frac{f_{j,i}^p (\partial \mu_j / \partial \beta_q + \partial \eta_j / \partial \beta_q) \min(d_{j,i}^p, d_{k,i}^p)}{\sum_{l \in p^p(i,k) \cap p^p(i,l)} f_{l,i}^p \min(d_{l,i}^p, d_{k,i}^p)} \tag{18}$$
$$+ \delta_{pq} \sum_{k \in p^p(i,k) \cap p^p(i,j)} f_{k,i}^p \mu_k \frac{f_{j,i}^p (1 - \mu_j - \eta_j) \min(d_{j,i}^p, d_{k,i}^p)}{\sum_{l \in p^p(i,k) \cap p^p(i,l)} f_{l,i}^p \min(d_{l,i}^p, d_{k,i}^p)} \},$$

$$\frac{\partial \alpha_i^p}{\partial F_{\gamma\gamma'}^q} = \sum_j \{ f_{j,i}^p \partial \mu_j / \partial F_{\gamma\gamma'}^q + \sum_{k \in p^p(i,k) \cap p^p(i,j)} f_{k,i}^p (\partial \mu_k / \partial F_{\gamma\gamma'}^q) \beta_p \frac{f_{j,i}^p (1 - \mu_j - \eta_j) \min(d_{j,i}^p, d_{k,i}^p)}{\sum_{l \in p^p(i,k) \cap p^p(i,l)} f_{l,i}^p \min(d_{l,i}^p, d_{k,i}^p)}$$
$$- \sum_{k \in p^p(i,k) \cap p^p(i,j)} f_{k,i}^p \mu_k \beta_p \frac{f_{j,i}^p (\partial \mu_j / \partial F_{\gamma\gamma'}^q + \partial \eta_j / \partial F_{\gamma\gamma'}^q) \min(d_{j,i}^p, d_{k,i}^p)}{\sum_{l \in p^p(i,k) \cap p^p(i,l)} f_{l,i}^p \min(d_{l,i}^p, d_{k,i}^p)}$$
$$+ \delta_{pq} f_{j,i}^{q,\gamma\gamma'} \mu_j + \delta_{pq} \sum_{k \in p^q(i,k) \cap p^q(i,j)} f_{k,i}^{q,\gamma\gamma'} \mu_k \beta_q \frac{f_{j,i}^q (1 - \mu_j - \eta_j) \min(d_{j,i}^q, d_{k,i}^q)}{\sum_{l \in p^q(i,k) \cap p^q(i,l)} f_{l,i}^q \min(d_{l,i}^q, d_{k,i}^q)}$$
$$+ \delta_{pq} \sum_{k \in p^q(i,k) \cap p^q(i,j)} f_{k,i}^q \mu_k \beta_q \frac{f_{j,i}^{q,\gamma\gamma'} (1 - \mu_j - \eta_j) \min(d_{j,i}^q, d_{k,i}^q)}{\sum_{l \in p^q(i,k) \cap p^q(i,l)} f_{l,i}^q \min(d_{l,i}^q, d_{k,i}^q)}$$
$$- \delta_{pq} \sum_{k \in p^q(i,k) \cap p^q(i,j)} f_{k,i}^q \mu_k \beta_q \frac{f_{j,i}^q (1 - \mu_j - \eta_j) \min(d_{j,i}^q, d_{k,i}^q)}{\left(\sum_{l \in p^q(i,k) \cap p^q(i,l)} f_{l,i}^q \min(d_{l,i}^q, d_{k,i}^q) \right)^2} \sum_{l \in p^q(i,k) \cap p^q(i,l)} f_{l,i}^{q,\gamma\gamma'} \min(d_{l,i}^q, d_{k,i}^q) \}. \tag{19}$$

After the above length calculations, we can now compute

$$\frac{\partial \Delta E_i^{in}}{\partial TR_\gamma} = (1 - TR_i) \sum_q \partial \alpha_i^q / \partial TR_\gamma - (i \in V_\gamma) \sum_q \alpha_i^q, \tag{20}$$

$$\frac{\partial \Delta E_i^{in}}{\partial \chi} = (1 - TR_i) \sum_q \partial \alpha_i^q / \partial \chi, \chi \neq TR_\gamma, \tag{21}$$

$$\frac{\partial \Delta R_i^{in}}{\partial TR_\gamma} = (1 - TR_i) \sum_{q,j} f_{j,i}^q \partial \eta_j / \partial TR_\gamma - (i \in V_\gamma) \sum_{q,j} f_{j,i}^q \eta_j, \tag{22}$$

$$\frac{\partial \Delta R_i^{in}}{\partial F_{\gamma\gamma'}^q} = (1 - TR_i) \sum_{q,j} f_{j,i}^q \partial \eta_j / \partial F_{\gamma\gamma'}^q + (1 - TR_i) \sum_{q,j} f_{j,i}^{q,\gamma\gamma'} \eta_j, \tag{23}$$

$$\frac{\partial \Delta R_i^{in}}{\partial \chi} = (1 - TR_i) \sum_{q,j} f_{j,i}^q \partial \eta_j / \partial \chi, \chi = \beta_q, z, R_0, D_I, D_E, \tag{24}$$

$$\frac{\partial \Delta S_i^{in}}{\partial TR_\gamma} = -(i \in V_\gamma) \sum_{q,j} f_{j,i}^q - \frac{\partial \Delta R_i^{in}}{\partial TR_\gamma} - \frac{\partial \Delta E_i^{in}}{\partial TR_\gamma}, \tag{25}$$

$$\frac{\partial \Delta S_i^{in}}{\partial F_{\gamma\gamma'}^q} = (1 - TR_i) \sum_{j} f_{j,i}^{q,\gamma\gamma'} - \frac{\partial \Delta R_i^{in}}{\partial F_{\gamma\gamma'}^q} - \frac{\partial \Delta E_i^{in}}{\partial F_{\gamma\gamma'}^q}, \tag{26}$$

$$\frac{\partial \Delta S_i^{in}}{\partial \chi} = -\frac{\partial \Delta R_i^{in}}{\partial \chi} - \frac{\partial \Delta E_i^{in}}{\partial \chi}, \chi = \beta_q, z, R_0, D_I, D_E. \tag{27}$$

In the end we arrive at computing gradients of S_i, E_i, I_i and R_i.
